# New Method of Treatment in Phthisis. III.—Cantharidinate of Potash

**Published:** 1891-10-17

**Authors:** 


### THE PRACTITIONER'S MIRROR AND RETROSPECT.

[*The Editor will be glad to receive offers of co-operation and contributions from members of the profession. All letters should be addressed to* THE EDITOR, THE LODGE, PORCHESTER SQUARE, LONDON, W.]

### MEDICINE.

### NEW METHOD OF TREATMENT IN PHTHISIS.

III.—CANTHARIDINATE OF POTASH.

On February 25th, 1891, Professor Oscar Liebreich brought before the Berlin Medical Society some results obtained in the treatment of phthisis of the larynx by the administration of cantharidin, and although he did not for a moment claim to have discovered a specific for tuberculous affections, yet very marked beneficial effects were said to have been obtained. But this like many of the newly-introduced methods, is but a modification or rather a new application, of an old remedy. It was employed by Hafeland in lung diseases with some good results, and Cazenauve claimed that psoriasis may be cured by tincture of cantharidin taken internally. We may even go further back, for cantharis is mentioned as a remedy by Hippocrates and Pliny. Cantharidin is derived from the Spanish fly, Lytha vesicatoria, and was isolated, in 1812, by Robiquet. It forms small colourless and odourless crystals, almost completely insoluble in water, slightly soluble in alcohol, and more so in ether and chloroform. Cantharidin seems to exercise an elective affinity for the healthy kidney. The action of cantharidin on the kidney was the subject of careful investigation by Cornil, and his results have been well known to pathologists. But the lungs, too, become much affected, in fact the fatal effect is stated to be the result of an exudation therein, which impedes respiration and causes dyspnœa. The consistence of the lungs appears increased, and microscopically the change appears to be not an acute œdema, but the effusion of an exudation poor in cells, with little tendency to coagulate. This outpour is due to a direct irritation of the capillaries from the chemical action of the cantharidin. The irritative action in the vascular wall may be greater if the vessel is no longer in a healthy condition, and may be induced by a dose too small to effect healthy capillaries. Now it is quite conceivable that the exudation of serum induced may be sufficient to revive the almost extinct energies of the cells which are affected by diseased processes, and that the freshly outpoured serum may exert bactericidal properties, as well as help the cells to exert their defensive powers. Professer Liebreich commenced his injections with doses of $\frac{1}{10}$ of a milligramme in a case of tumour of the œsophagus The injection caused no pain, nor any inflammatory changes at the seat of puncture, and the patient volunteered that expectoration was rendered more easy. Several patients in the Augusta Hospital under Drs. Ewald and Gunlich, Hahu, Bode, and others, were subjected to this treatment. The dose was gradually raised in several cases until six decimilligrammes were given, when symptoms of effects upon the uro-genital tract were observed. These, however, soon disappeared after a few drops of the tincture of opium. It was found that the $\frac{1}{120}$ of a grain was the maximum dose. Cantharidin being insoluble in water, the solution for hypodermic use is made by the addition of some alkali, which renders it soluble. The most convenient is potassium or sodium hydrate, of which 0·4 gramme is taken, with 0·2 gramme of cantharidin; the mixture is dissolved over a water bath in 1,000 c.cm. of water. One c.cm. of this solution (equal to two decimilligrammes of the substance) is injected into the back. The patient is allowed to follow his employment, but attention is paid to the state of the urine and bowels. The injection may be given on alternate days; but if unfavourable symptoms occur, the dose is reduced by one-half.

The results of the necropsy in two patients treated by cantharidin are reported by Luebimoff, of Kazan. In one fifteen, and in the other twenty injections had been given. In both, the lung, spleen, liver, and kidneys were found studied with enormous masses of tubercle of the usual appearance. Neither the number of the tubercle bacilli, nor their morphological properties and behaviour towards staining substances, showed any deviation from what is observed in ordinary cases of phthisis. In both instances unmistakeable acute glomerulo-nephritis was detected, and in one the urinary tubules contained hyaline cases.

Dr. Heyman has used cantharidinate of potassium in twenty-seven cases, but of these seventeen only were satisfactory. All were suffering from laryngeal tuberculosis, with lung complications, and in all tubercle bacilli had been found in the sputum. They were treated as out-patients, and they continued their ordinary avocations. In a few cases a certain amount of diarrhœa was produced, and in some slight scalding on micturition. In the majority of the cases the hoarseness disappeared or was lessened, the patients felt better, the physical signs in the chest improved.

Fraenkel has reported the results in fifteen cases of laryngeal phthisis. He found that redness and suppuration of the vocal chords steadily diminished under the treatment, pain and swelling also disappeared, while the ulcers gradually became smaller, and, in some instances, healed entirely. Aphonia, which had been present before, disappeared in most cases.

In four cases of pulmonary tuberculosis under Drs. Tchaüsoff and Elsenberg, no amelioration was observed. Rosenbach has never observed any improvement of the local conditions in laryngeal phthisis in the cases in which he has used it. It has been employed in five cases by Forlanini, of Turin. Three of these were phthisis pulmonalis, one was a case of pleurisy, probably tuberculous, and one croupous pneumonia. In two cases no appreciable result was noticed, in the other three more harm resulted than good. Forlanini concludes that injections of cantharidin are followed by a certain amount of both general and local reaction analogous to that which follows the use of tuberculin. In all three cases the injections were followed by albuminuria and urobilinuria. Cornil has tried Liebreich's treatment in seven patients suffering from laryngo-pulmonary tuberculosis. In no case was there the slightest improvement, and in one there was considerable œdema of the ary-epiglottic folds, and of the connective tissue at the base of the tongue; this, M. Cornil does not think it fair to attribute to the injections, as the condition not infrequently becomes developed in the ordinary course of the disease. On the whole, the beneficial action of cantharidin does not seem to him to be proved either in laryngeal or in pulmonary tuberculosis. He believes, moreover, that the prolonged use of the remedy, even in small doses, may produce serious lesions of the kidney, and his prolonged investigations as to the pathological action of cantharis in the kidney entitle his views on the subject to our most careful consideration.

We have referred to the results obtained by several observers, from which it will be gathered that the treatment is by no means devoid of danger, and that it is extremely doubtful whether the beneficial effects reported by some of the Berlin experimenters, were really attributable to the remedy.

REFERENCES.—*Therap. Gaz.*, Apr. 15, 1891; *Prac.*, June, 1891; *Berl. Klin. Woch.*, No. 9, 1891; *Brit. Med. Journ.* Supp., May 2—Aug. 15, 1891; *Vratch*, No. 17, 1891, p. 434; *Gazetta. Med.*, Lombard, May 9, 1891; *Journ. des Conaisses. Méd.*, Ap. 16, 1891.

